# One-year test-retest reliability of ten vision tests in Canadian athletes

**DOI:** 10.12688/f1000research.19587.5

**Published:** 2020-09-09

**Authors:** Mehdi Aloosh, Suzanne Leclerc, Stephanie Long, Guowei Zhong, James M. Brophy, Tibor Schuster, Russell Steele, Ian Shrier

**Affiliations:** 1 Department of Epidemiology, Biostatistics and Occupational Health, McGill University, Montreal, Canada; 2 Department of Health Research Methods, Evidence, and Impact, Michael G. DeGroote School of Medicine, McMaster University, Hamilton, Canada; 3 Institut National du Sport du Quebec, Montreal, Canada; 4 Department of Family Medicine, McGill University, Montreal, Canada; 5 Faculty of Medicine, McGill University, Montreal, Canada; 6 Department of Mathematics and Statistics, McGill University, Montreal, Canada; 7 Centre for Clinical Epidemiology, Lady Davis Institute, Jewish General Hospital, McGill University, Montreal, Canada

**Keywords:** concussion, vision tests, binocular, saccades, reliability

## Abstract

**Background**: Vision tests are used in concussion management and baseline testing. Concussions, however, often occur months after baseline testing and reliability studies generally examine intervals limited to days or one week. Our objective was to determine the one-year test-retest reliability of these tests.

**Methods**: We assessed one-year test-retest reliability of ten vision tests in elite Canadian athletes followed by the Institut National du Sport du Quebec. We included athletes who completed two baseline (preseason) annual evaluations by one clinician within 365±30 days. We excluded athletes with any concussion or vision training in between the annual evaluations or presented with any factor that is believed to affect the tests (e.g. migraines). Data were collected from clinical charts. We evaluated test-retest reliability using Intraclass Correlation Coefficient (ICC) and 95% limits of agreement (LoA).

**Results:** We examined nine female and seven male athletes with a mean age of 22.7 (SD 4.5) years. Among the vision tests, we observed excellent test-retest reliability in Positive Fusional Vergence at 30cm (ICC=0.93) but this dropped to 0.53 when an outlier was excluded in a sensitivity analysis. There was good to moderate reliability in Negative Fusional Vergence at 30cm (ICC=0.78), Phoria at 30cm (ICC=0.68), Near Point of Convergence break (ICC=0.65) and Saccades (ICC=0.61). The ICC for Positive Fusional Vergence at 3m (ICC=0.56) also decreased to 0.45 after removing two outliers. We found poor reliability in Near Point of Convergence (ICC=0.47), Gross Stereoscopic Acuity (ICC=0.03) and Negative Fusional Vergence at 3m (ICC=0.0). ICC for Phoria at 3m was not appropriate because scores were identical in 14/16 athletes. 95% LoA of the majority of tests were ±40% to ±90%.

**Conclusions:** Five tests had good to moderate one-year test-retest reliability. The remaining tests had poor reliability. The tests would therefore be useful only if concussion has a moderate-large effect on scores.

## Introduction

Concussion, a form of mild traumatic brain injury is a growing public health concern ^1^. Estimates suggest up to 3.8 million sport-related concussions occur annually in the United States, with 50% going unreported ^2^. United States emergency department visits for sports-related traumatic brain injuries have increased 60% over 2001–2009 ^3^. Concussions can be associated with headaches, dizziness, visual disturbances, and other symptoms that can negatively affect performance in sport, school, and work and negatively impact quality of life ^2, 4, 5^.

Diagnosis of concussion and decisions to return-to-play are based on symptoms, signs, physical examination and special tests ^6^. Previous research has shown an association between concussion and eye movement ^1^. Concussion may therefore affect multiple aspects of vision, including saccades, pursuit, convergence, accommodation, and vestibulo-ocular reflex ^7^. Some studies reported 50% to 90% incidence of visual symptoms, such as blurred vision and diplopia in individuals with concussion ^8^. Therefore, vision testing may be helpful in the assessment and management of patients with concussion.

Each vision test measures a function that is linked to a particular brain structure or pathway. Vision tests are noninvasive tests with rapid administration and scoring. Understanding test variability, independent of changes in pathology or recovery (i.e. reliability), is required to assess their clinical utility. However, only a limited number of reliability studies have assessed binocular vision tests and saccades ^9– 20^. In addition, these reliability studies measured a specific aspect of the vision. These studies are not uniform in their method and they are diverse in their population.

Previous investigations of the test-retest reliability of these vision tests have used short test-retest time intervals ranging from 0 to approximately 57 days ^9– 20^, except for one test of saccades ^21^. For test-retest reliability to be useful in clinical management (e.g. return-to-play), the time intervals must reflect the time frame in which they would be used ^22^. The previous studies have provided information on the usefulness of these tests when following improvement or deterioration of patients over short periods of time. However, concussions usually occur several months and up to one year after annual baseline testing, and not as 0 days to 57 days as in the previous studies. Therefore, we examined one-year test-retest reliability of ten vision tests in Canadian athletes over one year period of time.

## Methods

### Participants

The study population included athletes over 16 years of age followed by the Institut National du Sport du Quebec (INSQ) in Canada from 2015–2018. Many of these athletes had a yearly examination done by a sports medicine physician and vision tests done by a clinician trained in orthoptic testing.

We only included athletes who had completed two baseline (preseason) annual evaluations within a 365-day (± 30 days) time period. We excluded athletes who suffered a concussion in between annual evaluations or had received preventive orthoptic training between the baseline measures. We also excluded athletes with a history of strabismus or treated strabismus, or were medically treated for depression, anxiety or psychiatric conditions that may affect binocular vision and saccades. Data were collected from electronic medical charts of one clinician trained in orthoptic measures and one sports medicine physician.

### Measures

At the beginning of each season, athletes underwent baseline testing of ten vision tests by a single orthoptic-trained clinician (industry partner). The vision tests were Gross Stereoscopic Acuity, Near Point of Convergence (NPC), Near Point of Convergence break (NPCb), near (30cm) and far (3m) Positive Fusional Vergence, near (30cm) and far (3m) Negative Fusional Vergence, near (30cm) and far (3m) Phoria, and Saccades.

A detailed description of each test including the procedures of each test and the theoretical range of scores is provided in Table 1. We will briefly describe each vision test here. We used a horizontal prism bar with the base-out for Positive Fusional Vergence and base-in for Negative Fusional Vergence, at both 30cm and 3m ^10^. Phoria was measured at 30cm and 3m using the prism and alternate cover test using the procedures described by the Pediatric Eye Disease Investigator Group ^23^. To perform NPC and NPCb, we followed the Maples *et al*., protocol ^13^. We measured Gross Stereoscopic Acuity with the Randot Stereotest (Stereo Optical Co., Inc., Chicago, IL) according to the manufacturer’s instructions ^24^. Evaluation of Saccades was done using the test procedures developed by the orthoptic-trained clinician. Participants assumed a tandem stance an arm’s length away from a screen attempting to fixate on appearing and disappearing lights on the screen, while trying to keep their head still. Light flashes appeared at a rate of 100 per minute for two minutes. This test was scored by the clinician based on quality (bad, medium, good), synchronization (bad, medium, good), and saccadic corrections (many, few, none). These three components were then combined into an overall percentage saccade score, based on an unpublished proprietary algorithm developed by the clinician who performed the testing.

**Table 1.** Detailed description of the ten vision tests.

**Positive Fusional** **Vergence**	This test examines how well a participant can adapt to challenges in fixating light on their retina at near distance (30cm) and far distance (3m), measured in prism diopters. The seated participant fixates on a fixed target at the appropriate distance. The clinician begins by using the weakest prism strength (base-out) which forces the participant to converge their eyes to maintain fixation. The strength of the prism is increased until the participant can no longer maintain a single image. The score of each test (30cm and 3m) is the strength of the prism in which the participant maintained binocular vision, with higher scores representing better function. The range of normative data for Positive Fusional Vergence at near fixation is 35 to 40 prism diopters, and the range at far fixation is 16 to 20 prism diopters ^25– 27^.
**Negative Fusional** **Vergence**	This is the same test as Positive Fusional Vergence except the horizontal prism bar is positioned base-in, forcing the participant to diverge their eyes to maintain fixation on a fixed object positioned at near (30cm) and far (3m), measured in prism diopters. The clinician incrementally increases the strength of the prism until the participant is no longer able to maintain a single image. The score of each test is the strength of the prism in which the participant maintained binocular vision, with higher scores representing better function. The range of normative data for Negative Fusional Vergence at near fixation is 12 to 16 prism diopters, and the range at far fixation is 6 to 8 prism diopters ^25– 27^.
**Phoria**	We evaluated the natural deviation of the eyes (heterophoria), in prism diopters, with the prism and alternate cover test using a target placed at (1) 3m from the participant (far vision), and (2) 30cm from the participant (near vision). While the seated participant was fixating on the target, the clinician covered and uncovered each of the participant’s eyes to trigger movements while using a prism bar (base-out if the eye moves outward, base-in if the eye moves inward) to cancel these movements. The prism power was progressively increased until no shift in the eyes was seen. The score of the test was the rating of the prism that canceled the eye movements, with lower scores representing less Phoria. We were unable to find normative data for this test.
**Near Point of** **Convergence (NPC)**	NPC assesses the ability to symmetrically converge, and is sometimes referred to as “motor punctum proximum” ^26^, in cm. The seated participant fixates on a near target 30cm away. The target is gradually moved towards their eyes as they attempt to maintain fixation. NPC is reached when one or both eyes can no longer maintain fixation on the target, which is identified as when one eye diverges outwards. The score of the test is the distance (cm) between the bridge of the nose and the distance of the target at the closest point at which the individual could maintain balanced oculomotor synergy between both eyes. Lower scores indicate better NPC. Normative data in older textbooks report average NPC values for healthy adults between 6 to 8 cm ^28^, but a more recent study suggested 5 cm should be considered the upper limit of normal values ^29^.
**Near Point of** **Convergence break** **(NPCb)**	This test is conducted using the same methods as NPC, but the test ends when the participant has double vision due to the inability of the eyes to converge. The score of the test is the distance between the bridge of the nose and the point (in cm) where double vision occurs, where a lower score indicates better NPCb. Normative data for elementary school children with normal vision suggested a mean of 3.3 cm, with a range of 1.0 to 13.7 cm ^30^; however, data on adults with normal vision suggest a breakpoint of approximately 5.0 to 7.5 cm ^31^.
**Gross Stereoscopic** **Acuity**	We tested the ability to perceive depth with the Randot® Stereotest (Stereo Optical Co., Inc., Chicago, IL), in arc seconds. Seated participants wearing polarized glasses were asked to hold the testing booklet 16 inches from their face. Participants were then presented images formed of dots that are displaced in relation to each other. The test steadily increased in difficulty by reducing the level of disparity between dots, beginning at 400 arc seconds (lowest possible score) and ending at 20 arc seconds (highest possible score). A participant’s score was the arc seconds corresponding to the smallest disparity at which the participant identified the raised (i.e. stereoscopic) image. Normative data suggest the average score for an adult is 40 arc seconds ^32, 33^.
**Saccades**	This test examines the eye’s ability to perform saccadic movements, which are rapid eye movements that abruptly alter the point of fixation. In our clinician’s version of this test, participants assume a tandem stance (heel-to-toe with dominant foot in the back) standing an arm’s length away from the screen. Lights appear and disappear in different locations on the screen at a rate of 100 flashes per minute, for a total of two minutes. The participant is instructed to keep their head still and only move their eyes to fixate on the appearing lights. The clinician observes the eyes for quality and synchronization (rated: bad, medium, good) and saccadic correction (rated: many corrections, few corrections, no corrections). The three sub- scores were combined into an overall percentage score according to a proprietary algorithm developed by the clinician (industry partner) who performed the testing. There are no normative data for this version of the test because the score is based on a proprietary algorithm.

### Analysis

We report the mean (SD) for continuous variables at baseline. We evaluated test-retest reliability using Intraclass Correlation Coefficient (ICC) ^34^ and 95% limits of agreement (LoA) ^35^. We considered ICC of ≤0.5 as poor, 0.51–0.74 as moderate, 0.75–0.89 as good, and ≥0.90 as excellent reliability ^36^. We report the LoA in the raw units of the scale used by clinicians. To compare LoA across tests, we also standardized the scores and reported them as percent differences, [(T1- T2)/ mean(T1&T2)]*100 ^35, 37^. Additionally, we summarized LoA graphically with Bland-Altman plots for each vision test using the standardized score for the y-axis to provide an overview of all vision tests. The raw scale measures are provided in parentheses to provide clinicians with information for individual patient assessment. Finally, we conducted a sensitivity analysis for the vision tests by excluding outliers that may have augmented the ICC results. We defined an outlier as a data point that was 1.5 interquartile ranges below the first quartile or above the third quartile.

Due to the limited sample size (n=16) and to avoid being overly conservative in our evaluation, we followed the practical solution for addressing multiple testing proposed by Saville, the unrestricted least significant difference procedure (or multiple t-test) ^38^. Formal multiplicity correction of confidence levels was not performed but we thoroughly reported all statistical assessments enabling an informal type-I error assessment by the reader. The data were analyzed using R statistical software 3.4.3 ^39^. This study was approved by the McGill University Faculty of Medicine Institutional Review Board.

## Results

**Figure 1.**
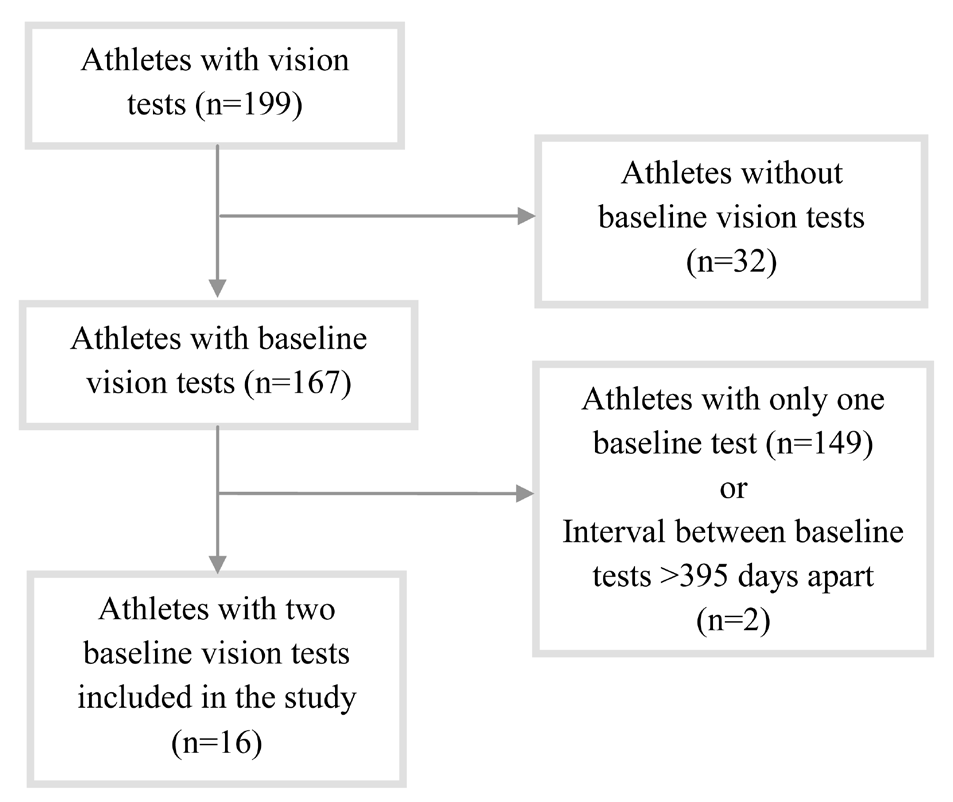
Patient flow diagram.

Of the 199 athletes measured for the vision tests, only 16 individuals met our inclusion criteria ( Figure 1). There were nine female and seven male athletes with a mean age of 22.7 (4.5) years at the baseline (preseason) measurement. Participants were athletes of water polo (n=6) and short-track speed skating (n=10). A second measurement was conducted between 335 and 372 days (mean of 356.4 (17.3) days) after the initial baseline.

**Figure 2.**
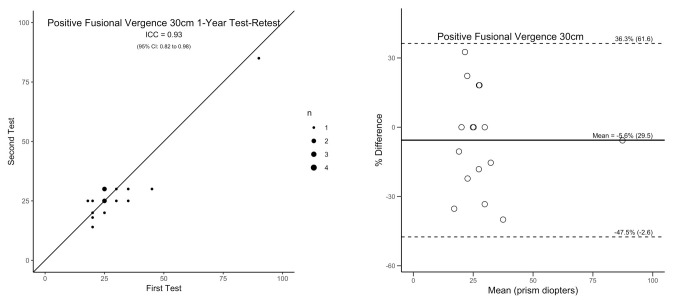
Vision test with excellent one-year test-retest reliability. ( **A**) Scatter plot of test-retest reliability for Positive Fusional Vergence at 30cm. Identity line represents perfect agreement between the test-retest values; ICC refers to the Intraclass correlation coefficient and 95%CI refers to the 95% Confidence Interval. “n (1,2,3,4)” refers to the number of participants represented by each dot when scores exactly overlapped. ( **B**) Bland-Altman plot with the mean of the test-retest on the x-axis and the difference between test-retest on the y-axis. Solid line represents the bias and dotted lines represent the 95% LoA. The y-axis represents a standardized LoA using percentage difference on the plot to allow one to compare the different tests to each other. The LoA in the units of measure, which are familiar to clinicians, are provided in the parentheses. When the analysis was repeated excluding the outlier to the far right, the ICC decreased to 0.53 and the 95% LoA increased to 43.5%.

The range of scores observed for each vision test can be found in each of the reliability figures ( Figure 2– Figure 4) ^40^. Our analysis suggested one-year test-retest reliabilities ranging from poor to excellent among the ten vision tests. Including all the data, we observed excellent one-year test-retest reliability in Positive Fusional Vergence at 30cm with ICC of 0.93 ( Figure 2). In this test, 4 out of 16 pairs of measurements were identical after 1 year. The range of measurements was between 14 and 45 diopters with one outlier at 90 diopters. LoA of the test was ±41.9%. Given the very high ICC and the presence of an outlier that greatly increased the range of the values for the measure (known to increase ICC), we repeated the analysis excluding the outlier. This decreased the ICC from 0.93 to 0.53, and increased the LoA to ±43.5%. One of the reviewers for this paper has insisted that the analysis without the outlier be considered the primary analysis.

**Figure 3.**
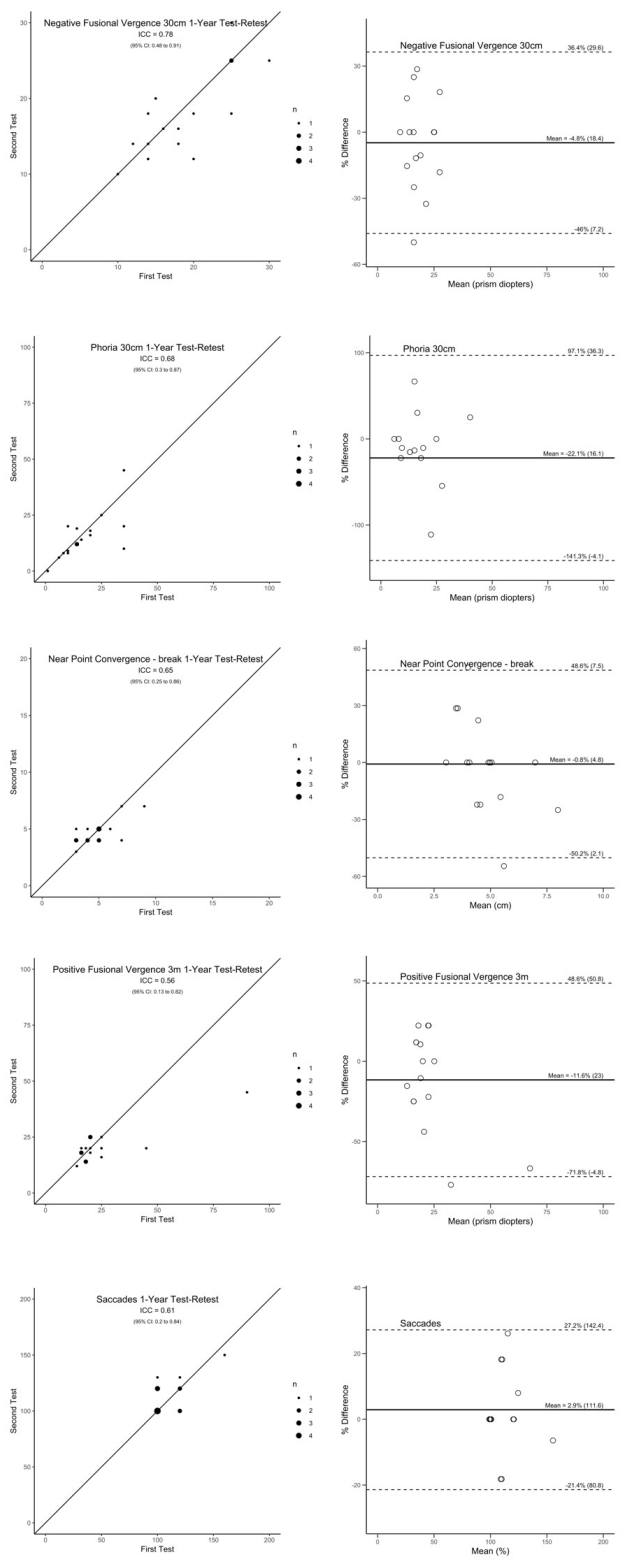
Vision tests with good to moderate one-year test-retest reliability. ( **A**) Scatter plot of test-retest for Negative Fusional Vergence at 30cm, Phoria at 30cm, Near Point of Convergence break (NPCb), Positive Fusional Vergence at 3m, and Saccades. ( **B**) Bland-Altman plot related to each test. See Figure 2 for explanation of abbreviations and scales. When the analysis for Positive Fusional Vergence at 3m was repeated excluding the two outliers, the ICC decreased to 0.45 and the 95% LoA decreased to 41.4%.

**Figure 4.**
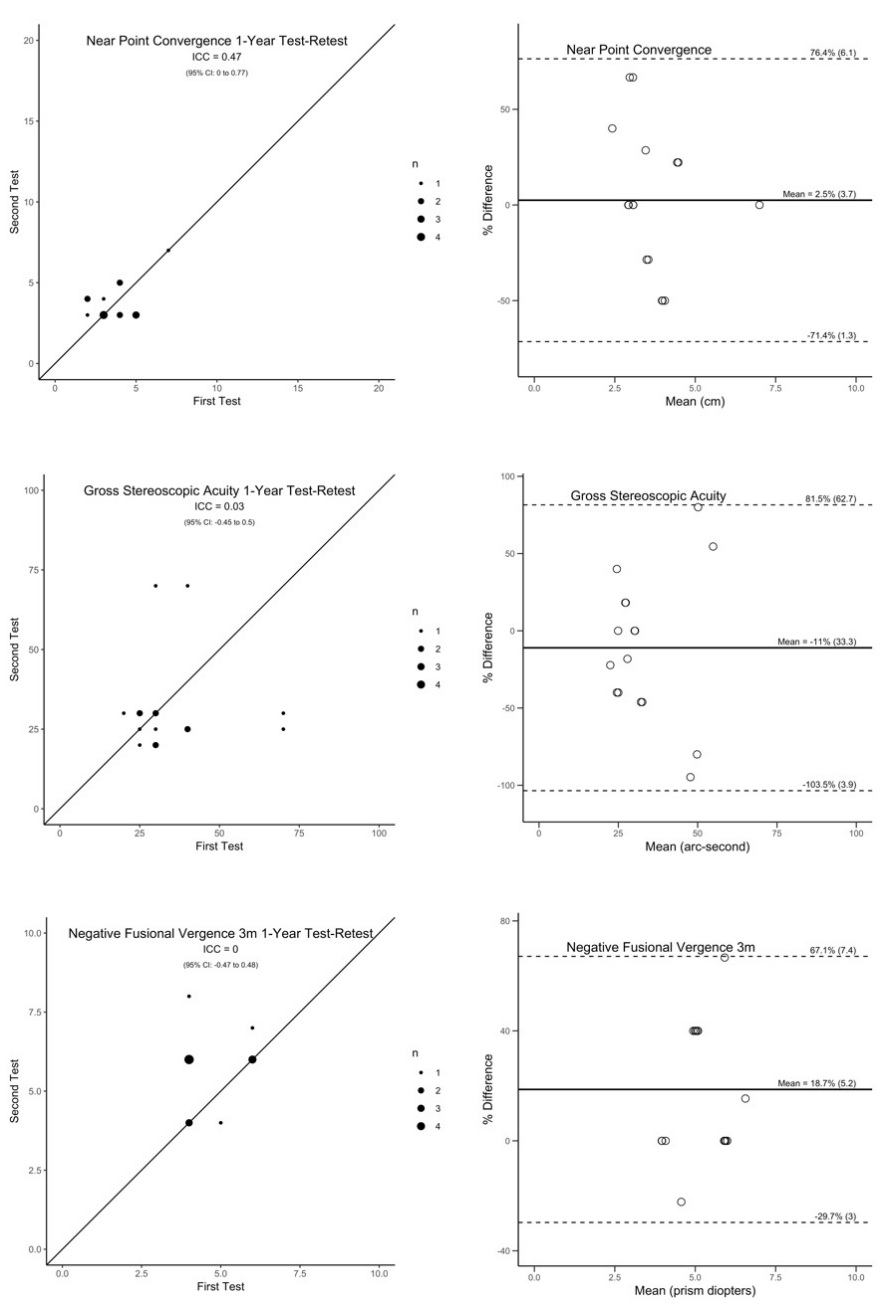
Vision tests with poor one-year test-retest reliability. ( **A**) Scatter plots of test-retest for near point of convergence (NPC), Gross Stereoscopic Acuity, and Negative Fusional Vergence at 3m. ( **B**) Bland-Altman plots related to each test. See Figure 2 for explanation of abbreviations and scales.

Five tests showed good to moderate one-year test-retest reliability ( Figure 3), including Negative Fusional Vergence at 30cm (ICC=0.78, LoA=41.2%), Phoria at 30cm (ICC=0.68, LoA=119.2%), NPCb (ICC=0.65, LoA=49.4%), Positive Fusional Vergence at 3m (ICC=0.56, LoA=60.2%), and Saccades (ICC=0.61, LoA=24.3%). There were two outliers for Positive Fusional Vergence at 3m (one participant on both measures and one participant on only one measure). When we removed both of these outliers, the ICC dropped from 0.56 to 0.45 and the 95% LoA decreased from 60.2% to 41.4%. In both of these cases, the two scores from the outlier were quite different. Although one might anticipate that the ICC would increase by removing such outliers, the ICC actually decreased because the range of values for the measure decreased substantially. As above, one of the reviewers for this paper insisted that the analysis without the outliers be considered the primary analysis.

Three of the remaining four tests showed poor one-year test-retest reliability ( Figure 4). These include NPC (ICC=0.47, LoA=73.9%), Gross Stereoscopic Acuity (ICC=0.03, LoA=92.5%) and Negative Fusional Vergence at 3m (ICC=0.0, LoA=48.4%). For Phoria at 3m, 14/16 athletes had identical scores on the two measures. In this context, the ICC and LoA were not appropriate measures of reliability and are not presented.

## Discussion

We found that the one-year test-retest reliability for 10 vision tests in young elite athletes ranged from moderate to poor after accounting for outliers. The majority of the vision tests had standardized 95% LoA in the range of 40–90%, which indicates that repeated scores of an individual over time may vary by 40–90% of the mean score even without any actual change in vision function.

There are a limited number of test–retest reliability studies on non-vision neurocognitive tests over a one year period in teenage athletes. For instance, the ICC for different components of Immediate Post-Concussion Assessment and Cognitive Testing (ImPACT), a computerized brain injury measurement tool, ranges from 0.50 to 0.82 ^41^. However, we could not find any research examining the stability of the vision tests over a one year period, in athlete or non-athlete populations except for one test of saccades that was very different from the test used in this study ^21^. It is important that test-retest reliabilities fall within a range needed for clinical interpretation of concussion assessment and for discussion about return-to-play. In the context of comparing results after a concussion to annual baseline tests conducted in the pre-season, the time-frame for reliability comparisons should be up to one year ^22^.

Although there are no long-term reliability studies on the ten vision tests evaluated in this study, a number of studies have reported short term test-retest reliability of individual tests using various methods among various groups of individuals, including children and healthy adults ^9– 20^. Using NPC as a general example, one study reported excellent immediate test-retest reliability in concussed athletes (ages 9–24) (ICC = 0.95 to 0.98) ^12^. A separate study using a 2–3 day test-retest protocol found the ICC = 0.65 for NPC in healthy individuals (calculated in Rouse *et al.*, 2002 ^16^ for data from reference ^15^), and a third study reported one week test-retest ICC = 0.89 and 0.92 for NPCb in healthy school children ^16^.

We recently examined one-week test-retest reliability of the same ten vision tests with the same methods and same age-range as this current study in 20 young non-athletes. We found one-week test-retest reliability ranging from poor (ICC = 0.34) to good (ICC = 0.88), with five out of ten tests showing moderate reliability (ICCs = 0.54 to 0.69) ^17^. This suggests that these vision tests can only be useful if a concussion has a moderate to large effect on scores. Overall, the ICCs in the current study were generally smaller than those reported in our one-week study, suggesting increased temporal variability. Unexpectedly, the 95% LoA for one-year test-retest was smaller or equal to the 95% LoA of the one-week test-retest for all vision tests except NPC (±73.9 vs. ±57.9) and Gross Stereoscopic Acuity (±92.5 vs. ±55). In addition, in both the one-week and one-year intervals, almost all individuals had the same value in Phoria 3m, which leads to uninformative LoA.

In one-year test-retest, Positive Fusional Vergence showed excellent reliability at 30cm (ICC=0.93) and moderate at 3m (ICC=0.56), initially. Our results at 30cm were significantly better than those of another study examining test-retest reliability of Positive Fusional Vergence at 30cm in children (ICCs of 0.53–0.59) ^16^. Perhaps more importantly, our results were also better than the one-week test-retest reliability conducted by the same clinician with the same methods in our previous prospective research study (ICC=0.54 and 0.49, respectively) ^17^. It is difficult to understand how test-retest reliability over one year could be better than test-retest reliability over one week. When we explored the data further, we noticed one outlier that greatly increased the range of values for Positive Fusional Vergence at 30cm ( Figure 2) and Positive Fusional Vergence at 3m ( Figure 3). Increasing the range of values is known to increase the ICC. This is because ICC is based on the results of an analysis of variance which separates the error into variability between individuals (range of values along x or y axes) and variability within an individual. Therefore, if variability between persons increases, indicated by a larger range of values, ICC will increase. When we removed the outlier for Positive Fusional Vergence at 30cm, the ICC dropped to 0.53, which is similar to the value found for the one-week test-retest reliability (ICC=0.54); the LoA increased to 43.5%. When we removed the two outliers from Positive Fusional Vergence at 3m, the ICC decreased to 0.45 and LoA decreased to 41.4%. Note that the outliers for this measure had large differences between the two test scores, and removing such data points would normally be expected to increase the ICC ( Figure 3). The finding that the ICC decreased indicates that as expected, if the range of values among the populations is similar, the one-year test-retest reliability for Positive Fusional Vergence at both 30cm and 3m is likely less than the one-week test-retest reliability.

In addition to Positive Fusional Vergence, two other tests also had higher ICC at one year (Negative Fusional Vergence 30cm: 0.78 vs 0.66) and Saccades (0.61 vs 0.34) but there were no apparent outliers and the range of values were similar in the two studies. Aside from outliers, there are other theoretical reasons that might explain why ICC is better at one-year than at one-week. First, it is possible that the non-athletes in our one-week test-retest study had less motivation to perform well on the repeat tests. If true, their scores would be less than the motivated athletes performing during the one-year test-retest. Second, there is a potential learning effect in retest measurements that could affect results. A learning effect, however, is unlikely in our study because the athletes were tested only twice, with a one-year interval between tests. Third, the one-week study was a prospective research study where the clinician performing the test was blinded. Our current results are based on clinical charts where the clinician had access to the previous results which might artificially increase the reliability of the test. Fourth, the increased ICC could have occurred simply by chance because of sampling variation.

Our measurements of Phoria at 30cm had moderate reliability for near (ICC=0.68) consistent with our one-week retest reliability study (ICC=0.69) ^17^. Other studies in adults and children with strabismus ^42^ or esotropia ^23^ have not reported ICC. Therefore, comparing between studies is not possible. Moreover, our analytical methods differed slightly from those studies. We evaluated all angles of deviation together, and other authors analyzed smaller (2–20 Prism Diopter) or larger (>20 Prism Diopter) angles of strabismus separately because of different prism increments measured ^42^. For Phoria at 3m, we found that the ICC and LoA were not appropriate measures of reliability because most of the population reported identical scores of zero for both measurements. One may consider that if we had a wider range of scores, ICC might provide meaningful information.

One-year test-retest reliability of NPC and NPCb (0.47 and 0.65, respectively) were similar to the results in our one-week reliability study (0.54 and 0.64, respectively) ^17^. Brozek *et al*. found a similar ICC of 0.65 for NPC in healthy adults (calculated in Rouse *et al.*, 2002 ^16^ for data from Brozek *et al.*, 1948 ^15^). However, Giffard *et al.* reported a one-week ICC = 0.84 in patients for NPC with neck pain ^18^ and Rouse *et al.* reported excellent one-week reliability for NPCb in school children (ICC=0.89 and 0.92 for two different examiners) ^16^. The discrepancies in results are most likely due to differences in testing procedures. For instance, we used the Maples method ^13^ which is a non-accommodative test. Rouse *et al.* ^16^ used an accommodative target with Astron International Accommodative Rule and Giffard *et al.* ^18^ used the RAF rule ^28^.

Our one-year test-retest results for Gross Stereoscopic Acuity in young athletes showed poor reliability (ICC=0.03; 95% LoA= ±92.5%) even though our previous one-week test-retest results reported good reliability in non-athlete young adults (ICC=0.86; 95% LoA = ± 54%) ^17^ and another study using Titmus stereo fly and Frisby stereo tests in pre-school children revealed an excellent one-week reliability (ICC=1.0) ^19^. In addition, another study reported that 82.0% of their participants had identical results at test and retest taken on the same day in 100 healthy adult and children ^11^. With a one-year ICC of 0.03 and LoA of 92.5%, Gross Stereoscopic Acuity cannot be considered a reliable test to assess the vision function over one year, although it may still be appropriate for use in shorter time intervals, such as one week ^11, 17, 19^.

Finally, our clinician’s test of Saccades showed moderate reliability (ICC=0.61) with the smallest LoA (in percentage) of other tests, similar to the one-week study ^17^. These results are similar to other findings in healthy adults over a two-month period (ICC=0.59) ^20^. With a moderate reliability and the smallest LoA amongst the other vision tests, the results of the test of Saccades could be considered stable over a one year period assessing athletes.

In this study, four vision tests (Negative Fusional Vergence at 30cm, Phoria at 30cm, Saccades and NPCb) had moderate one-year test-retest reliability. The one test with identical scores in 14/16 athletes was Phoria at 3m. Therefore we cannot comment on the reliability of this test. This level of reliability would be useful in conditions where the concussion leads to a moderate change in vision function. The remaining five vision tests, including Positive Fusional Vergence at 30cm and 3m, NPC, Negative Fusional Vergence at 3m, and Gross Stereoscopic Acuity may be useful to detect the effect of concussion with a large change on vision function. Further studies are therefore required to assess the effect of concussion on vision test scores of the five vision tests. If it can be shown that the concussion has moderate to large effect on the test scores then these vision tests may still be useful clinically.

### Strengths and limitations

Several studies have previously evaluated the inter-rater reliability of some vision tests ^23, 42^. However, inter-rater reliability is less important in the context of clinical care when patients are followed by one clinician over time. Our study evaluated the test-retest reliability of the ten vision tests over an interval that allows for the normal variation over time expected in clinical practice between baseline measures and subsequent concussions. The ICC represents how much of variability in scores is due to differences between subjects. For instance, the ICC of 0.78 for near Negative Fusional Vergence at 30cm suggests that 78% of the variability in the measurements was due to differences between participants, and 22% was due to normal variations within the measurement. Furthermore, the 95% LoA for each test in our study provides the magnitude of the normal variation that can be expected with repeated measurements. Differences in test results between baseline and diagnosis of a concussion likely represent a true signal of a change in vision function within the patient if these differences are larger than the noise (LoA). In addition, we conducted sensitivity analysis to evaluate the effect of outliers. This analysis suggested that our initial ICC results may have been artificially high for two tests. (Positive Fusional Vergence at 30cm and 3m). Finally, the results of the test of Saccades in this study are based on the unpublished proprietary algorithm developed by the clinician. This limits its applicability for other clinicians.

This is a historical cohort observational study, a study design which has inherent limitations. The data provided were not always as precise as one might expect (e.g. near point convergence measured to the nearest cm). Some data in these athletes appear to be outside the normative range of data previously described for the general population. Because the data were obtained as part of clinical practice, the clinician had access to the results of the first test when conducting the repeat test one year later. The lack of blinding may result in higher agreement between the two tests compared to our blinded one-week research study. However, clinicians are not blinded during normal clinical practice, and therefore the results of this study would represent an expected level of agreement in that context, even if some of the agreement is due to bias. In addition, the sample size was relatively small and composed of healthy athletes, which will limit the generalizability of these findings to other populations. Although we started with a pool of 199 athletes, many athletes were excluded because they only had one baseline test, a concussion occurred in between the two baseline tests, or the second baseline test occurred outside the testing window of 365±30 days. Despite starting with athletes from many sports, only athletes from water polo and short-track speed skating met our eligibility criteria. It is unclear if subconcussion impacts affect neurological function in general ^43^. If subconcussion impacts were common in these sports and affected vision testing, we should have seen a systematic decrease in vision capacity between the two tests; this was not observed. Further, if it were present, the effect would be considered part of the “noise” clinicians have to consider when comparing the results from post-concussion and baseline tests. With an effective sample size of 16, the anticipated precision of ICC estimates was +/- 0.25 and the study had 80% power to detect ICC values >= 0.6 and more than 90% power to detect ICC values >=0.7 i.e. rejection of the null hypothesis (Table 1a in 44). Note that a total of >60 individuals were required to exclude ICC values <=0.5 with 80% power and an anticipated true ICC>0.7 (Table 2b in 44).

## Conclusion

We found that five out of the ten vision tests (Negative Fusional Vergence at 30cm, Phoria at 30cm, NPCb, Positive Fusional Vergence at 30cm, and Saccades) had good to moderate one-year test-retest reliability. This level of reliability is useful in conditions which produce a moderate change in vision function. The remaining five vision tests may be useful in detecting large effects on vision function. If further studies suggest that the effect of concussion on test scores is moderate to large, these vision tests may still be useful clinically.

## Data availability

Open Science Framework: Vision Tests in Concussion. https://doi.org/10.17605/OSF.IO/VB4W8 ^40^

Data are available under the terms of the Creative Commons Attribution 4.0 International license (CC-BY 4.0).

Demographic data are not available. With only 9 males and 7 females from our clinical source, any demographic information would immediately allow some participants to be identified and therefore this information cannot be shared in order to preserve participant confidentiality.
