# Supplementary material for: One-year test-retest reliability of ten vision tests in Canadian athletes
Source: F1000Res. 2020 Sep 9;8:1032. Originally published 2019 Jul 9. [Version 5] doi: 10.12688/f1000research.19587.5 (PMC7484722; doi:10.12688/f1000research.19587.5)
Supplement: Peer review history

# Peer review: Reviewer response for version 5

**DOI:** 10.5256/f1000research.29392.r71042

**Published:** 2020-09-10

**Authors:** James P Dickey, Dillon Richards

**Affiliations:** 1 School of Kinesiology, University of Western Ontario, London, Ontario, Canada; 2 Health and Rehabilitation Sciences, University of Western Ontario, London, Canada

The authors have revised the paper to  acknowledge "Some data in these athletes appear to be outside the normative range of data previously described for the general population", and have provided access to the raw data through the data availability link. This enables the readers to evaluate the credibility fo the data and interpret the findings accordingly.

Is the work clearly and accurately presented and does it cite the current literature?

Yes

If applicable, is the statistical analysis and its interpretation appropriate?

Yes

Are all the source data underlying the results available to ensure full reproducibility?

Yes

Is the study design appropriate and is the work technically sound?

Partly

Are the conclusions drawn adequately supported by the results?

Partly

Are sufficient details of methods and analysis provided to allow replication by others?

Partly

Reviewer Expertise:

Biomechanics, head impact exposure in sports, concussion

We confirm that we have read this submission and believe that we have an appropriate level of expertise to confirm that it is of an acceptable scientific standard.

# Peer review: Reviewer response for version 4

**DOI:** 10.5256/f1000research.28661.r70272

**Published:** 2020-09-02

**Authors:** James P Dickey, Dillon Richards

**Affiliations:** 1 Health and Rehabilitation Sciences, University of Western Ontario, London, Canada; 2 School of Kinesiology, University of Western Ontario, London, Ontario, Canada

Thank you for considering the points that we raised in the review, and we note that your recent revisions better characterize the effects of the outliers. We note that you have not chosen to acknowledge our point that these data points are extreme outliers (four data points at 3 or more IQRs above the third quartile, including one value 8.125 IQRs above the third quartile), and that they exceed the range of normative data for Positive Fusional Vergence that you present in Table 1. Our previous review stated that there is a strong reason for believing that these data points are questionable. In fact there is strong evidence that these data points should be eliminated rather than simply evaluating their influence in a "sensitivity analysis".

Is the work clearly and accurately presented and does it cite the current literature?

Yes

If applicable, is the statistical analysis and its interpretation appropriate?

Yes

Are all the source data underlying the results available to ensure full reproducibility?

Yes

Is the study design appropriate and is the work technically sound?

Partly

Are the conclusions drawn adequately supported by the results?

Partly

Are sufficient details of methods and analysis provided to allow replication by others?

Partly

Reviewer Expertise:

Biomechanics, head impact exposure in sports, concussion.

We confirm that we have read this submission and believe that we have an appropriate level of expertise to confirm that it is of an acceptable scientific standard, however we have significant reservations, as outlined above.

# Response

**Published:** 2020-09-03

**Authors:** Ian Shrier

**Affiliations:** Department of Family Medicine, McGill University, Canada

**Responses to reviewer 2&3:**

**Comment:** Thank you for considering the points that we raised in the review, and we note that your recent revisions better characterize the effects of the outliers. We note that you have not chosen to acknowledge our point that these data points are extreme outliers (four data points at 3 or more IQRs above the third quartile, including one value 8.125 IQRs above the third quartile), and that they exceed the range of normative data for Positive Fusional Vergence that you present in Table 1. Our previous review stated that there is a strong reason for believing that these data points are questionable. In fact there is strong evidence that these data points should be eliminated rather than simply evaluating their influence in a “sensitivity analysis”.

**Response: **We thank the reviewers for their feedback. There are three points raised.

1. The reviewers insist that results excluding outliers be considered the primary analysis, and the results including all the data be considered secondary.
2. The reviewers suggest there are four outliers instead of the 2 outliers we noted.
3. The reviewers suggest that 4 points exceed the range of normative data we provided.

1. Sensitivity Analyses

We think that there are different ways to look at outliers and interpret the results. As we mentioned in our previous response to the reviewers, we think that “eliminating” data, as the reviewers suggest, is not the optimal approach. Instead, the sensitivity analysis, as we performed, is a preferable approach unless there is a clear data error. In this way, we are transparent about our data set and our analysis, and the readers can evaluate our findings. F1000Research does not have an editor as an arbitrator when authors and reviewers disagree. Therefore, we have made the changes recommended by the reviewer and the analysis with the excluded data is now considered the primary result. We had already based our conclusions on the analyses after exclusions and have now edited the rest of the text as well.

2. Outliers

We are not sure why the reviewer thinks there are four outliers. The data in our study are available online at https://osf.io/gnjdm/. Here are the calculations for outliers, which we defined using the common standard: 1.5*IQR above the 3 ^rd^ quantile.

Positive Fusional Vergence 30cm

First Test:

25%: 20

75%: 31.25

IQR: 11.25

1.5*IQR: 16.9

Outlier Threshold (75%+1.5*IQR): 48.2

Second Test

25%: 23.75

75%: 30

IQR: 6.25

1.5*IQR: 9.4

Outlier Threshold (75%+1.5*IQR): 39.4

There is only one person with values that should be considered as outliers for positive fusional vergence at 30cm (Id=14). This occurred for both tests (90 on the first test and 85 on the second test). The text now reads:

- “Given the very high ICC and the presence of an outlier that greatly increased the range of the values for the measure (known to increase ICC), we repeated the analysis excluding the outlier. This decreased the ICC from 0.93 to 0.53, and increased the LoA to ±43.5%. One of the reviewers for this paper has insisted that the analysis without the outlier be considered the primary analysis.”

We also added a sentence to the figure legend:

- “When the analysis was repeated excluding the outlier to the far right, the ICC decreased to 0.53 and the LoA increased to 43.5%.”

Positive Fusional Vergence 3m

First Test

25%: 17.5

75%: 25

IQR: 7.5

1.5*IQR: 11.3

Outlier Threshold (75%+1.5*IQR): 36.3

Second Test

25%: 17.5

75%: 21.25

IQR: 3.75

1.5*IQR: 5.6

Outlier Threshold (75%+1.5*IQR): 26.8

There are two people with values that should be considered as outliers for positive fusional vergence at 3m (Ids 14 and 15). Particiant 14 is an outlier for both measures, and Particicpant 15 is an outlier for the first test. When Participant 14 was removed the ICC dropped from 0.56 to 0.21 as we reported. If we remove only Participant 15, the ICC actually increases from 0.56 to 0.63. If we remove both outliers, the ICC was 0.45 and the LoA decreased from 60.2% to 41.4%. As per the reviewer’s request, we are reporting the analysis with both outliers removed. The text now reads:

- “There were two outliers for Positive Fusional Vergence at 3m (one participant on both measures and one participant on only one measure). When we removed both of these outliers, the ICC dropped from 0.56 to 0.45 and the LoA decreased from 60.2% to 41.4%. In both of these cases, the two scores from the outlier were quite different. Although one might anticipate that the ICC would increase by removing such outliers, the ICC actually decreased because the range of values for the measure decreased substantially. As above, one of the reviewers for this paper insisted that the analysis without the outliers be considered the primary analysis.”

We have also added a sentence to the figure legend that says:

- “When the analysis for Positive Fusional Vergence at 3m was repeated excluding the two outliers, the ICC decreased to 0.45 and the LoA decreased to 41.4%.”

The results of these tests are also reported in the Discussion. The text in that section now reads:

- “When we removed the outlier for Positive Fusional Vergence at 30cm, the ICC dropped to 0.53, which is similar to the value found for the one-week test-retest reliability (ICC=0.54); the LoA increased to 43.5%. When we removed the two outliers from Positive Fusional Vergence at 3m, the ICC decreased to 0.45 and LoA decreased to 41.4%. Note that the outliers for this measure had large differences between the two test scores, and removing such data points would normally be expected to increase the ICC ( Figure 3). The finding that the ICC decreased indicates that as expected, if the range of values among the populations is similar, the one-year test-retest reliability for Positive Fusional Vergence at both 30cm and 3m is likely less than the one-week test-retest reliability.”

4. Normative Data Range

We do not know why some data were outside previously described normative data range. These are the data we received from the clinician doing the test as part of his regular clinical practice. It is possible that previously published normative data for the population does not represent normative data for athletes like those included in our study. We have added one sentence mentioning this in the limitations section of the article. It says:

- “Some of the data in these athletes appear to be outside the normative range of data previously described for the general population.”

# Peer review: Reviewer response for version 4

**DOI:** 10.5256/f1000research.28661.r70273

**Published:** 2020-08-27

**Authors:** M Nadir Haider

**Affiliations:** 1 Jacobs School of Medicine and Biomedical Sciences, State University of New York at Buffalo, Buffalo, NY, USA

No additional comments.

Is the work clearly and accurately presented and does it cite the current literature?

Yes

If applicable, is the statistical analysis and its interpretation appropriate?

Yes

Are all the source data underlying the results available to ensure full reproducibility?

No source data required

Is the study design appropriate and is the work technically sound?

Yes

Are the conclusions drawn adequately supported by the results?

Partly

Are sufficient details of methods and analysis provided to allow replication by others?

Yes

Reviewer Expertise:

Concussion, biomarkers, physiology, cerebral blood flow.

I confirm that I have read this submission and believe that I have an appropriate level of expertise to confirm that it is of an acceptable scientific standard.

# Peer review: Reviewer response for version 3

**DOI:** 10.5256/f1000research.27076.r66529

**Published:** 2020-07-20

**Authors:** James P Dickey, Dillon Richards

**Affiliations:** 1 Health and Rehabilitation Sciences, University of Western Ontario, London, Canada; 2 School of Kinesiology, University of Western Ontario, London, Ontario, Canada

This is an interesting and important paper based on the prevalence of concussion and the growing appreciation vision tests for diagnosing and assessing concussion. 

 The outlying data points in Positive Fusional Vergence at 30 cm and 3 m have been identified by the previous external peer reviewers and warrant additional consideration. You present your ICC findings with and without the outlying data points, which is appropriate. However, you do not fully characterize the extreme deviance of the outlying data points. Your wording about the outliers is rather misleading – you state “we noticed one outlier that greatly increased the range of values along x-axis in Figure 2 and Figure 3”. However, both the x- and y-coordinates of the outlier in Figure 2 meet your definition of outlier (1.5 interquartile ranges below the first quartile or above the third quartile), so it is not merely an issue with the x-axis. As well, you state “There was also one outlier for Positive Fusional Vergence at 3 m, 1.5 interquartile range above the third quartile”, but the 1.5 interquartile range (IQR) is your threshold for identifying outliers, not the description of the outlier – this data point is actually 8.125 IQRs above the third quartile. Statistically speaking, it is extremely unlikely that this data point is part of the same distribution as the rest of the data set. Finally, and perhaps most importantly, you state that you “have no reason to believe the data are inaccurate”. However, these outlying data points all exceed the range of normative data for Positive Fusional Vergence that you present in Table 1, providing a strong reason for believing that these data points are questionable. Your previous response “if deleting a point improved the ICC, we are confident the reviewer would agree that we should not delete the data point” trivializes the issue. The issues about the outlier data points must be more thoroughly addressed in the manuscript.

 It is highly unfortunate that the sample size is so limited, particularly since it would appear that your inclusion criteria were quite broad (followed by the Institut National du Sport du Quebec from 2015–2018). Examination of the participants' durations between tests reveals that the majority of the participants had 335-336 or 371-371 days between assessments - presumably these dates correspond to the timing of the preseason tests for the different sports. Would you have more eligible participants if you had broadened the eligibility criterion?

 It is unclear how it could be that your participants were limited to waterpolo and short-track speed skating, when presumably you started with a larger number of sports, but this should be clarified as it may reflect a bias in participant selection. As well, both waterpolo (Black *et al.* 2017) ^1^ and short-track speed skating (Quinn *et al.* 2003) ^2^ have a relatively high rate of concussions, and presumably the athletes may have received subconcussive head impacts, without receiving a concussion. Repetitive hits to the head are associated with microstructural and functional changes in the brain (Mainwaring *et al.* 2018) ^3^, and therefore should be acknowledged as a potential factor for the participants in this paper.

 You identify that test-retest reliability of vision tests has been evaluated at the 1 day to 45 days time span. However, studies have evaluated longer-term test-retest reliability. For example, Klein and Fischer (2005) ^4^ evaluated 19-month test–retest correlations of pro- and anti-saccadic eye movements on 117 participants. Of more direct relevance to the student athletes evaluated in your paper, Breedlove *et al.* (2019) ^5^ evaluated the reliability of the King-Devick test (prosaccades) on NCAA athletes, including 833 participants with measures one year apart, and Naidu *et al.* (2018) ^6^ evaluated the season-to-season reliability of the King-Devick Test in Canadian professional football players. Your paper would be strengthened by incorporating a fuller complement of relevant papers that have performed longer-term test-retest reliability measures of vision tests, and comparing your findings with theirs.

 The saccade measures reported in the paper have extremely limited value as they were collected using proprietary equipment - they should likely be removed from the paper.

 The scatterplots (Figures 2A, 3A and 4A) show the line of identity, but it would be interesting to also see the line of best fit. Furthermore, for the parameters with outliers, it would be interesting to add the lines of best fit with and without the outlier.

 The raw data presented through the Data Availability link is very helpful for gaining insight into the specifics of your data. However, it reveals that all of the data are reported as integers. Is this level of precision adequate for capturing the various vision tests? It would be helpful to include a "data dictionary", as recommended for best practices with spreadsheets (Broman and Woo, 2017) ^7^.

Is the work clearly and accurately presented and does it cite the current literature?

Yes

If applicable, is the statistical analysis and its interpretation appropriate?

Yes

Are all the source data underlying the results available to ensure full reproducibility?

Yes

Is the study design appropriate and is the work technically sound?

Partly

Are the conclusions drawn adequately supported by the results?

Partly

Are sufficient details of methods and analysis provided to allow replication by others?

Partly

Reviewer Expertise:

Biomechanics, head impact exposure in sports, concussion.

We confirm that we have read this submission and believe that we have an appropriate level of expertise to confirm that it is of an acceptable scientific standard, however we have significant reservations, as outlined above.

## References

[1] : The Epidemiology of Concussions: Number and Nature of Concussions and Time to Recovery Among Female and Male Canadian Varsity Athletes 2008 to 2011. *Clin J Sport Med*.2017;27(1) : 10.1097/JSM.0000000000000308 52-56 10.1097/JSM.0000000000000308 26862834

[2] : Injuries in short track speed skating. *Am J Sports Med*.31(4) : 10.1177/03635465030310040501 507-10 10.1177/03635465030310040501 12860536

[3] : Subconcussive head impacts in sport: A systematic review of the evidence. *Int J Psychophysiol*.132(Pt A) : 10.1016/j.ijpsycho.2018.01.007 39-54 10.1016/j.ijpsycho.2018.01.007 29402530

[4] : Instrumental and test-retest reliability of saccadic measures. *Biol Psychol*.2005;68(3) : 10.1016/j.biopsycho.2004.06.005 201-13 10.1016/j.biopsycho.2004.06.005 15620790

[5] : King-Devick Test Reliability in National Collegiate Athletic Association Athletes: A National Collegiate Athletic Association-Department of Defense Concussion Assessment, Research and Education Report. *J Athl Train*.2019;54(12) : 10.4085/1062-6050-219-18 1241-1246 10.4085/1062-6050-219-18 31618072PMC6922566

[6] : Sideline Concussion Assessment: The King-Devick Test in Canadian Professional Football. *Journal of Neurotrauma*.2018;35(19) : 10.1089/neu.2017.5490 2283-2286 10.1089/neu.2017.5490 29421971

[7] : Data Organization in Spreadsheets. *The American Statistician*.2018;72(1) : 10.1080/00031305.2017.1375989 2-10 10.1080/00031305.2017.1375989

# Response

**Published:** 2020-08-06

**Authors:** Ian Shrier

**Affiliations:** Department of Family Medicine, McGill University, Canada

**Author Responses**

**REVIEWER # 2&3**

**Comment:** This is an interesting and important paper based on the prevalence of concussion and the growing appreciation vision tests for diagnosing and assessing concussion. 

**Answer:** We thank the reviewers for their interest in our manuscript.

__________________

**Comment: **The outlying data points in Positive Fusional Vergence at 30 cm and 3 m have been identified by the previous external peer reviewers and warrant additional consideration. You present your ICC findings with and without the outlying data points, which is appropriate. However, you do not fully characterize the extreme deviance of the outlying data points. Your wording about the outliers is rather misleading – you state “we noticed one outlier that greatly increased the range of values along x-axis in Figure 2 and Figure 3”. However, both the x- and y-coordinates of the outlier in Figure 2 meet your definition of outlier (1.5 interquartile ranges below the first quartile or above the third quartile), so it is not merely an issue with the x-axis. As well, you state “There was also one outlier for Positive Fusional Vergence at 3 m, 1.5 interquartile range above the third quartile”, but the 1.5 interquartile range (IQR) is your threshold for identifying outliers, not the description of the outlier – this data point is actually 8.125 IQRs above the third quartile. Statistically speaking, it is extremely unlikely that this data point is part of the same distribution as the rest of the data set. Finally, and perhaps most importantly, you state that you “have no reason to believe the data are inaccurate”. However, these outlying data points all exceed the range of normative data for Positive Fusional Vergence that you present in Table 1, providing a strong reason for believing that these data points are questionable. Your previous response “if deleting a point improved the ICC, we are confident the reviewer would agree that we should not delete the data point” trivializes the issue. The issues about the outlier data points must be more thoroughly addressed in the manuscript.

**Answer: **We thank the reviewers for raising this point and have modified the text accordingly. For the comment that 1.5 IQR is the threshold and not the data point, we agree and have removed the phrase. It now reads:

“There was also one outlier for Positive Fusional Vergence at 3m. When removing this outlier in a sensitivity analysis, the ICC dropped from 0.57 to 0.21.”

The paragraph in which this is mentioned refers to the fact that the 1-year test-retest reliability had higher ICC than the 1-week test-retest reliability and this should not be possible. Our sensitivity analyses were conducted to determine if this occurred because of the increased range observed in the 1-year data. The reviewers are correct that the outlier in question is indeed an outlier on both the x and y axis. We have modified the text accordingly. This particular section now reads as below. Similar changes were made to other parts of the manuscript where appropriate:

“Given the very high ICC and the presence of an outlier that greatly increased the range of values for the measure (known to increase ICC), we conducted a sensitivity analysis excluding the outlier.”

With respect to justifying keeping the outlier in the plot or not, we did not mean to trivialize the issue. We only meant that the decision to remove an outlier needs more justification than simply that the data point was unexpected. Therefore, although we agree with the reviewers that our sensitivity analysis for the ICC is more likely to be correct, we do not feel there is enough evidence to replace the original analysis with the sensitivity analysis as the primary analysis. We feel that discussing this at length would be more confusing than helpful and have deleted the phrase related to “have no reason to believe the data are inaccurate”. The full paragraph now reads:

“In one-year test-retest, Positive Fusional Vergence showed excellent reliability at 30cm (ICC=0.93) and moderate at 3m (ICC=0.56), initially. These values were better than the one-week test-retest reliability (ICC=0.54 and 0.49, respectively) ^17^ . It is difficult to understand how test-retest reliability over one year could be better than test-retest reliability over one week. When we explored the data further, we noticed one outlier that greatly increased the range of values for Positive Fusional Vergence at 30cm (Figure 2) and Positive Fusional Vergence at 3m (Figure 3). Increasing the range of values is known to increase the ICC. This is because ICC is based on the results of an analysis of variance which separates the error into variability between individuals (range of values along x or y axes) and variability within an individual. Therefore, if variability between persons increases, indicated by a larger range of values, ICC will increase. We explored how removing the outlier in our data would affect the results. When we removed the outlier for Positive Fusional Vergence at 30cm, the ICC dropped to 0.53, which is below the value found for the one-week test-retest reliability; it did not affect LoA. When we removed the outlier (same person) from Positive Fusional Vergence at 3m, the ICC decreased to 0.21. Note that the outlier for this measure had a large difference between the two test scores, and removing such a data point would normally be expected to increase the ICC ( Figure 3). The finding that the ICC decreased indicates that as expected, if the range of values among the populations is similar, the one-year test-retest reliability for Positive Fusional Vergence at both 30cm and 3m is likely less than the one-week test-retest reliability.”

__________________

**Comment:** It is highly unfortunate that the sample size is so limited, particularly since it would appear that your inclusion criteria were quite broad (followed by the Institut National du Sport du Quebec from 2015–2018). Examination of the participants' durations between tests reveals that the majority of the participants had 335-336 or 371-371 days between assessments - presumably these dates correspond to the timing of the preseason tests for the different sports. Would you have more eligible participants if you had broadened the eligibility criterion?

**Answer: **We thank the reviewers for this comment. Our eligibility criteria only required that the athlete not have a concussion or undergo vision training between tests, and did not have a condition that would affect the results of vision testing. We are not sure which of these criteria the reviewers think we could relax and still obtain an unbiased answer to the question of 1-year test-retest reliability. We could have shortened the interval to only several months, but that would no longer be answering the 1-year test-retest reliability question. We have not made any changes to the manuscript.

__________________

**Comment:** It is unclear how it could be that your participants were limited to waterpolo and short-track speed skating, when presumably you started with a larger number of sports, but this should be clarified as it may reflect a bias in participant selection. As well, both waterpolo (Black et al. 2017) and short-track speed skating (Quinn et al. 2003) have a relatively high rate of concussions, and presumably the athletes may have received subconcussive head impacts, without receiving a concussion. Repetitive hits to the head are associated with microstructural and functional changes in the brain (Mainwaring et al. 2018), and therefore should be acknowledged as a potential factor for the participants in this paper.

**Answer: **We thank the reviewers for raising these points. For the types of sports participants were engaged in, these are the data provided to us. Many athletes from other sports only had 1 test, and some had concussions or vision testing within the 1-year interval. We do not have data on which athletes were referred for testing but never went for the test. The reviewers suggested Black et al reported waterpolo as a sport with many concussions. However, the study cited actually reported 0 concussions in waterpolo athletes. The Quinn et al study reported 6 concussions in 63 athletes over a 1-year period. The authors did not include the injury rate in the paper and it is not possible to compare risks to other sports without knowing how often the athletes were competing / practicing. In general, short-track speed skating concussions occur because of collisions that cause the athlete to fall, and then they may hit their head into the padded boards or on the ice. There are not multiple small hits like one would receive in American football or hockey. That said, we expand on the issue below for other studies that might include athletes from these types of sports.

The reviewers suggest cumulative subconcussive head impacts should be raised as potential factor for participants in this study. We are not sure what the reviewers mean. We agree with the paper by Mainwaring et al. (2018) that the reviewers cited. Mainwaring et al states:

 

- “Both the research and conceptual understanding of this phenomenon are in their infancy”
- “the findings are equivocal regarding the effect of subconcussive impacts on the brain”
-  “Insufficient evidence was presented to conclude that repetitive head impacts are associated with neurocognitive impairment. It may be that neuropsychological assessment tools are not sufficiently sensitive to detect any subtle changes in cognitive function that emerge from subconcussive impacts, or that the neurocognitive changes are inconsequential, or follow neurophysiological changes or damage.”
- “Future research is needed to characterize the phenomenon in question.”

 

As an example, one study found that repetitive subconcussive head impacts over a single season do not appear to result in short-term neurologic impairment (see Gysland SM, Mihalik JP, Register-Mihalik JK, Trulock SC, Shields EW, Guskiewicz KM. *The relationship between subconcussive impacts and concussion history on clinical measures of neurologic function in collegiate football players*. Annals of biomedical engineering. 2012;40(1):14-22).

Aside from these results that do not support a decrease in neurocognitive function with subconcussive impacts, our objective in this study was to report on the 1-year test-retest reliability of vision tests in order to help clinicians understand how to interpret differences between testing conducted post-concussion and at baseline. If subconcussive impacts did affect vision testing, one would expect a decline in visual function as a consequence of subconcussive impacts. If this occurred, any change in test scores between baseline and post-concussion could not be attributed to concussion. That said, we doubt this is the case because if that were true, one would expect a decline in vision function (and test scores) conducted one year after baseline. We did not observe this in our data. However, we acknowledge that the athletes in our study were not involved in sports with many subconcussive impacts. We have modified the text at the end of the limitation, which now includes:

“This is a historical cohort observational study, a study design which has inherent limitations. The data provided were not always as precise as one might expect (e.g. near point convergence measured to the nearest cm). In addition, the sample size was relatively small and composed of healthy athletes, which will limit the generalizability of these findings to other populations. Although we started with a pool of 199 athletes, many athletes were excluded because they only had one baseline test, a concussion occurred in between the two baseline tests, or the second baseline test occurred outside the testing window of 365±30 days. Despite starting with athletes from many sports, only athletes from Waterpolo and Short-track speed skating met our eligibility criteria. It is unclear if subconcussion impacts affect neurological function in general ^43^. If subconcussion impacts were common in these sports and affected vision testing, we should have seen a systematic decrease in vision capacity between the two tests; this was not observed. Further, if it were present, the effect would be considered part of the “noise” clinicians have to consider when comparing the results from post-concussion and baseline tests.”

__________________

**Comment: **You identify that test-retest reliability of vision tests has been evaluated at the 1-45 days time span. However, studies have evaluated longer-term test-retest reliability. For example, Klein and Fischer (2005) evaluated 19-month test–retest correlations of pro- and anti-saccadic eye movements on 117 participants. Of more direct relevance to the student athletes evaluated in your paper, Breedlove et al. (2019) evaluated the reliability of the King-Devick test (prosaccades) on NCAA athletes, including 833 participants with measures one year apart, and Naidu et al. (2018) evaluated the season-to-season reliability of the King-Devick Test in Canadian professional football players. Your paper would be strengthened by incorporating a fuller complement of relevant papers that have performed longer-term test-retest reliability measures of vision tests, and comparing your findings with theirs.

**Answer: **We thank the reviewers for the reference that we had not been aware of. Our study investigated tests for specific visual function. Although the King-Devick test is sometimes used in concussion, it measures a combination of functions much beyond visual function. Therefore, we do not feel it is relevant to our research questions. We were not aware of the Klein and Fischer article and have now included the reference in the Introduction and Discussion. Our test was quite different from that studied in Klein and Fisher. The introduction text now reads:

Previous investigations of the test-retest reliability of these vision tests have used short test-retest time intervals ranging from 1 day to 45 days ^9^ ^– 17^ , except for one test of saccades ^44^.

and the Discussion text now reads:

“However, we could not find any research examining the stability of the vision tests over a one year period, in athlete or non-athlete populations except for one test of saccades that was very different from the test used in this study ^44^.”

__________________

**Comment:** The saccade measures reported in the paper have extremely limited value as they were collected using proprietary equipment - they should likely be removed from the paper.

**Answer: **We respectfully disagree with the reviewers. First, we do not see any harm in including the result of a non-standard test and readers who are not interested can simply ignore the results. Second, we evaluated this non-standard test as this measure was in our *a priori* protocol. Omitting analyses described in an *a priori* protocol is a form of reporting bias that we would prefer to avoid. We have modified the limitation section to say:

“Finally, the results of the test of Saccades in this study are based on the unpublished proprietary algorithm developed by the clinician. This limits its applicability for other clinicians.”

__________________

**Comment:** The scatterplots (Figures 2A, 3A and 4A) show the line of identity, but it would be interesting to also see the line of best fit. Furthermore, for the parameters with outliers, it would be interesting to add the lines of best fit with and without the outlier.

**Answer: **We thank the reviewers for this comment. We believe the recommended statistical practice for evaluating reliability is the ICC with line of identity, and LOA. We have provided the references that guided this decision. Lines of best fit are not measures of reliability. In addition, any comparison of regression lines with the line of identity can be misleading because one must incorporate the uncertainty due to sampling. If the reviewers have an appropriate statistical reference that supports using regression in studies of test-retest reliability, we would be happy to add the analyses in a subsequent revision.

__________________

**Comment:** The raw data presented through the Data Availability link is very helpful for gaining insight into the specifics of your data. However, it reveals that all of the data are reported as integers. Is this level of precision adequate for capturing the various vision tests? It would be helpful to include a "data dictionary", as recommended for best practices with spreadsheets (Broman and Woo, 2017).

**Answer: **We thank the reviewers for their comment. We have developed a data dictionary and uploaded it as metadata. We agree that some of the measures could have been measured more precisely than others but these are the data provided from the clinician with expertise in orthoptics. We have added text to the beginning of the 2 ^nd^ paragraph in the limitations section which now reads:

“This is a historical cohort observational study, a study design which has inherent limitations. The data provided were not always as precise as one might expect (e.g. near point convergence measured to the nearest cm).”

# Peer review: Reviewer response for version 1

**DOI:** 10.5256/f1000research.21476.r60656

**Published:** 2020-03-10

**Authors:** M Nadir Haider

**Affiliations:** 1 Jacobs School of Medicine and Biomedical Sciences, State University of New York at Buffalo, Buffalo, NY, USA

Thank you for giving me the opportunity to review this manuscript. It measures the retest reliability of common ocular/oculomotor tests over one year. The sample size is 16 college-aged athletes. Intra-class correlation is performed and presented. I have read through the entire manuscript and it is exceptionally well-written, it shows that it has gone through several internal, and even some external, reviews and revisions already. The statistical analysis are correctly described and the appropriate tests and graphs are used to present data. 

 The most obvious downside of this study is the small sample size, there is so much within-subject variation among these test due to the natural process of aging and ocular adaptations which could be due to insignificant events like getting a new monitor for work. Future studies should be performed on larger sample sizes, etc.

 But I believe that there is merit in having your study indexed for a couple of reasons. The research protocol and analysis are well explained and could be used for design future oculomotor retest reliability studies. Secondly, I am glad that you had concussion as your exclusionary criteria since there are a hundred different publications showing abnormalities in vision function tests after concussion, yet present no retest reliability without the presence of a concussive head injury. I think this paper provides some preliminary evidence which should be made available to other researchers and I think this is a citable manuscript. I do not have any sentence by sentence suggestions, but my only major suggestion is to remove the pre-outlier ICC of Positive Fusional Vergence at 30cm value of 0.93 and say that it is 0.55 (moderate). And I think Negative Fusional Vergence at 30cm should be classified as Good ICC (not moderate since it is between 0.75 and 0.9).

Is the work clearly and accurately presented and does it cite the current literature?

Yes

If applicable, is the statistical analysis and its interpretation appropriate?

Yes

Are all the source data underlying the results available to ensure full reproducibility?

No source data required

Is the study design appropriate and is the work technically sound?

Yes

Are the conclusions drawn adequately supported by the results?

Partly

Are sufficient details of methods and analysis provided to allow replication by others?

Yes

Reviewer Expertise:

Statistical design, physiological and biochemical markers of concussion, autonomic regulation of cerebral blood blow.

I confirm that I have read this submission and believe that I have an appropriate level of expertise to confirm that it is of an acceptable scientific standard.

# Response

**Published:** 2020-03-23

**Authors:** Ian Shrier

**Affiliations:** Department of Family Medicine, McGill University, Canada

**REVIEWER #1**

**Comment:** Thank you for giving me the opportunity to review this manuscript. It measures the retest reliability of common ocular/oculomotor tests over one year. The sample size is 16 college-aged athletes. Intra-class correlation is performed and presented. I have read through the entire manuscript and it is exceptionally well-written, it shows that it has gone through several internal, and even some external, reviews and revisions already. The statistical analysis are correctly described and the appropriate tests and graphs are used to present data. 

**Answer:** We thank the reviewer for the kind comments.

________

**Comment:** The most obvious downside of this study is the small sample size, there is so much within-subject variation among these test due to the natural process of aging and ocular adaptations which could be due to insignificant events like getting a new monitor for work. Future studies should be performed on larger sample sizes, etc.

**Answer**: In this paper, we used all eligible participants from a clinical database. Therefore, we could not calculate an a priori sample size. Our primary approach to sample size requirements is to estimate precision rather than use hypothesis testing. We have tried to provide information for both approaches in the current version, and the new final paragraph of the limitations section is provided below.

"This is a historical cohort observational study, a study design which has inherent limitations. In addition, the sample size was relatively small and composed of healthy athletes, which will limit the generalizability of these findings to other populations. Although we started with a pool of 199 athletes, many athletes were excluded because they only had one baseline test, a concussion occurred in between the two baseline tests, or the second baseline test occurred outside the testing window of 365±30 days. With an effective sample size of 16, the anticipated precision of ICC estimates was  +/- 0.25 and the study had 80% power to detect ICC values >= 0.6 and more than 90% power to detect ICC values >=0.7 i.e. rejection of the null hypothesis (Table 1a in ^42^). Note that a total of  >60 individuals were required to exclude ICC values <=0.5 with 80% power and an anticipated true ICC>0.7 (Table 2b in ^42^)."

Reference: Bujang MA, N. B. A simplified guide to determination of sample size requirements for estimating the value of intraclass correlation coefficient: a review. *Arch Orofac Sci*. 2017; 12(1): 1-11.

______

**Comment:** But I believe that there is merit in having your study indexed for a couple of reasons. The research protocol and analysis are well explained and could be used for design future oculomotor retest reliability studies. Secondly, I am glad that you had concussion as your exclusionary criteria since there are a hundred different publications showing abnormalities in vision function tests after concussion, yet present no retest reliability without the presence of a concussive head injury. I think this paper provides some preliminary evidence which should be made available to other researchers and I think this is a citable manuscript.

**Answer**: We again thank the reviewer for the kind comments.

________

**Comment:** I do not have any sentence by sentence suggestions, but my only major suggestion is to remove the pre-outlier ICC of Positive Fusional Vergence at 30cm value of 0.93 and say that it is 0.55 (moderate).

**Answer:** We thank the reviewer for the comment. Recommended practice is to only delete data points if you have a very good reason to believe they are inaccurate. Otherwise, one should keep the original analysis intact and apply sensitivity analyses. For example, if deleting a point improved the ICC, we are confident the reviewer would agree that we should not delete the data point. For this reason, we have not changed our results as suggested. However, we have modified the text to further emphasize the importance of the sensitivity analysis.

_________

**Comment:** And I think Negative Fusional Vergence at 30cm should be classified as Good ICC (not moderate since it is between 0.75 and 0.9).

Answer: We thank the reviewer for pointing out this oversight. We have now indicated that Figure 3 shows results for good to moderate reliability tests, and made the associated changes in the abstract and manuscript as well.
